# Elevated C1orf63 expression is correlated with CDK10 and predicts better outcome for advanced breast cancers: a retrospective study

**DOI:** 10.1186/s12885-015-1569-2

**Published:** 2015-07-25

**Authors:** Chao-Qun Hong, Fan Zhang, Yan-Jie You, Wei-Li Qiu, Armando E. Giuliano, Xiao-Jiang Cui, Guo-Jun Zhang, Yu-Kun Cui

**Affiliations:** 1 Guangdong Provincial Key Laboratory for Breast Cancer Diagnosis and Treatment, Cancer Hospital of Shantou University Medical College, Shantou, 515041 China; 2 Department of pharmacy, Luohe Medical College, 148 Daxue-Road, Luohe, 462002 China; 3 Department of Surgery, Samuel Oschin Comprehensive Cancer Institute, Cedars-Sinai Medical Center, Los Angeles, CA 90048 USA

**Keywords:** C1orf63, CDK10, Overall survival, TNM stage

## Abstract

**Background:**

Chromosome 1 open reading frame 63 (C1orf63) is located on the distal short arm of chromosome 1, whose allelic loss has been observed in several human cancers. C1orf63 has been reported to be up-regulated in IL-2-starved T lymphocytes, which suggests it might be involved in cell cycle control, a common mechanism for carcinogenesis. Here we investigated the expression and clinical implication of C1orf63 in breast cancer.

**Methods:**

Paraffin-embedded specimens, clinicopathological features and follow-up data of the breast cancer patients were collected. Publicly available microarray and RNA-seq datasets used in this study were downloaded from ArrayExpress of EBI and GEO of NCBI. KM plotter tool was also adopted. The expression of C1orf63 and CDK10, one known cell cycle-dependent tumor suppressor in breast cancer, was assessed by immunohistochemistry. Western blotting was performed to detect C1orf63 protein in human breast cancer cell lines, purchased from the Culture Collection of the Chinese Academy of Sciences, Shanghai.

**Results:**

In a group of 12 human breast tumors and their matched adjacent non-cancerous tissues, C1orf63 expression was observed in 7 of the 12 breast tumors, but not in the 12 adjacent non-cancerous tissues (*P* < 0.001). Similar results were observed of C1orf63 mRNA expression both in breast cancer and several other cancers, including lung cancer, prostate cancer and hepatocellular carcinoma. In another group of 182 breast cancer patients, C1orf63 expression in tumors was not correlated with any clinicopathological features collected in this study. Survival analyses showed that there was no significant difference of overall survival (OS) rates between the C1orf63 (+) group and the C1orf63 (−) group (*P* = 0.145). However, the analyses of KM plotter displayed a valid relationship between C1orf63 and RFS (relapse free survival)/OS (*P* < 0.001; *P* = 0.007). Notablely, in breast cancers with advanced TNM stages (III ~ IV) among these 182 patients, C1orf63 expression was an independent prognostic factor predicting better clinical outcome (HR: 0.41; 95 % CI: 0.17 ~ 0.97; *P* = 0.042). Additionally, we found that CDK10 mRNA expression was positively correlated with C1orf63, which was consistent with the relationship of protein expression between C1orf63 and CDK10 (*r*_s_ = 0.391; *P* < 0.001).

**Conclusions:**

Compared to adjacent non-cancerous tissues, C1orf63 expression was elevated in tumor tissues. However, C1orf63 predicts better prognosis for breast cancers with advanced TNM stage, and the underlying mechanism is unknown. In addition, C1orf63 is correlated with the cell cycle related gene, CDK10.

## Background

The initiation and development of breast cancer is a multistep process encompassing progressive changes in genetic aberrations in normal tissue, resulting in hyperplasia with or without atypia, *in situ* carcinomas, invasive carcinomas, and finally metastatic carcinoma [1]. Increasing evidence reveals that molecular subtyping of this malignancy is crucial to better understand the clinical behavior of these tumors and to identify the targets for better therapy [2, 3].

Chromosome 1 open reading frame 63 (C1orf63), also known as arginine/serine-rich protein 1 (RSRP1, NCBI Gene ID: 57035), is located at 1p36.13 - p35.1. Although the function of C1orf63 is still unclear, frequent allelic loss on the distal short arm of chromosome 1 has been reported in a broad range of solid human tumors, including breast, non-small cell lung and colorectal cancers [4]. Especially, allelic loss at 1p31.1-36.3 was shown to be an early event in the carcinogenesis of breast cancer [5]. The allelic loss at 1p34-36 was demonstrated to be an independent predictor of shorter disease-free survival for patients with node-negative breast cancer [6]. Thus, these regions on 1p may harbor tumor suppressor genes [7]. Furthermore, it was reported that the transcription of C1orf63 was upregulated in the interleukin (IL)-2-dependent human T cells, which were forced to exit cell cycle by IL-2 withdrawal, indicating that C1orf63 could be involved in cell cycle exit and acted as a cellular quiescence-controlling gene. Its expression might represent one early event for tumorigenesis [8]. However, the involvement of C1or63 in the oncogenesis and progression of breast cancer has not been reported before.

In the current study, C1orf63 protein expression was detected in breast cancer tissues, and correlated to the clinicopathological features and prognosis of breast cancer. Then the relationship between C1orf63 and cyclin-dependent kinase 10 (CDK10), a known cell cycle-dependent tumor suppressor in breast cancer [9, 10] was investigated. Furthermore, the potential association between the expression of C1orf63 and known breast cancer biomarkers including estrogen receptor (ER), progesterone receptor (PR), and human epidermal growth factor receptor 2 (HER-2) were also examined.

## Methods

### Tumor samples and cell culture

Paraffin-embedded archival pathological specimens, complete clinicopathological features and follow-up data were retrieved for 182 breast cancer patients (women, median age: 51 years; range: 29–88 years). The patients had undergone curative surgery without preoperative therapy, at the Cancer Hospital of Shantou University Medical College, between October 2001 and November 2002. Clinical tumor stage (TNM stage) was grouped in accordance with the American Joint Committee on Cancer (AJCC) 6th Ed Cancer Staging Manual (2002). In this study, stages III and IV were designated as advanced stage, while stages I and II were early stage [11]. The clinicopathologic features for these patients, including expression status of ER, PR and HER-2, were summarized in Table 1. The corresponding adjacent normal tissues of 12 patients were also obtained from surgical resections. The observation period ranged from 1 to 159 months (the median period was 42 months). Informed consent for the use of their samples was obtained from all the patients. This study was approved by the medical ethics committee of the Cancer Hospital of Shantou University Medical College.

**Table 1:** Relationship of C1orf63 expression with clinicopathologic features and biomarkers. 182 patients with breast cancer were included and the correlations between C1orf63 expression and clinicopathologic features were analyzed using chi-square test

Clinicopathological features	C1orf63 expression		Chisq	*P*
	Negative (≤4) *n* = 138 (%)	Positive (>4) *n* = 44 (%)		
Age, year				
≤ 60	114 (74.5)	39 (25.5)	0.905	0.341
> 60	24 (82.8)	5 (17.2)		
T (Primary tumor)				
T0 ~ T2	81 (75.0)	27 (25.0)	0.183	0.669
T3 ~ T4	56 (77.8)	16 (22.2)		
N (Regional lymph nodes)				
N0 ~ N1	73 (78.5)	20 (21.5)	0.601	0.438
N3 ~ N4	64 (73.6)	23 (26.4)		
TNM stage				
I ~ II	58 (78.4)	16 (21.6)	0.315	0.575
III ~ IV	80 (74.8)	27 (25.2)		
ER				
Negative	55 (79.7)	14 (20.3)	1.115	0.291
Positive	80 (72.7)	30 (27.3)		
PR				
Negative	85 (79.4)	22 (20.6)	2.319	0.128
Positive	50 (69.4)	22 (30.6)		
HER-2				
Negative	82 (75.9)	26 (24.1)	0.061	0.804
Positive	52 (74.3)	18 (25.7)		

Four breast cancer cell lines used in this study, namely MCF-7, MDA-MB-231, SK-BR-3 and BT549, were purchased from the Culture Collection of the Chinese Academy of Sciences, Shanghai, and maintained in DMEM (high glucose) containing 5 % fetal bovine serum.

### Immunohistochemistry of breast tissues

Immunohistochemistry (IHC) for C1orf63 and CDK10 was carried out using a standard EnVision complex method [12]. Briefly, sections (4-μm) were fixed in 10 % buffered formalin and embedded in paraffin. After deparaffinization and rehydration, endogenous peroxidase activity was blocked with 0.3 % hydrogen peroxide for 30 min. Then tissue sections were autoclaved at 121 °C in citrate buffer (pH 6.0) for 10 min, and incubated with rabbit anti-C1orf63 polyclonal antibody (1:100 dilution, Beijing Biosynthesis Biotechnology Co., Ltd., China) or CDK10 antibody (1:300 dilution, Abgent, San Diego, USA). IHC staining was carried out by an EnVision antibody complex (anti-mouse/rabbit) method using an Envision™ Detection kit (ZSGB-BIO, Beijing, China) and 3,3’-diaminobenzidine as the chromogen substrate. A negative control was obtained by replacing the primary antibody with normal rabbit IgG.

IHC staining for C1orf63 was scored, as described [13] by a combination of intensity (0, no staining; 1, weak staining; 2, moderate staining; 3, strong staining) and proportion (0, < 5 % of tumor cells stained; 1, 5 - 25 % positive cells; 2, 26-50 % positive cells; 3, 51 - 75 % positive cells; 4, more than 76 % positive cells). If the product of multiplication between staining intensity and the proportion of positive cells was > 4, expression was defined as positive. Two pathologists independently assessed the cellular location and intensity of immunostaining in each section.

### Western blotting

Cells were lysed with a lysis buffer [50 mmol/L Tris–HCl (pH 8.0), 150 mmol/L NaCl, 1 % Triton X-100, and 100ug/ml PMSF] on ice for 30 min and centrifuged at 12000 rpm for 15 min at 4 °C. Cell lysates (20 ug) were electrophoresed on 10 % SDS-polyacrylamide gel and transferred onto a PVDF membrane. After blocking with Tris-buffered saline containing 0.05 % Tween 20 (TBST) and 5 % non-fat milk for 1 h at room temperature, the filters were washed 3 times/5 min with TBST and then incubated with antibodies against either anti rabbit C1orf63 (1:3000) or anti mouse actin (1:6000, Santa Cruz Biotechnology, Santa Cruz, USA) diluted in blocking buffer for 1 h, followed by incubation with horseradish peroxidase-labelled antirabbit (1:6000, Novus Biologicals, Littleton, USA) or antimouse (1:6000, Santa Cruz Biotechnology) IgG, and washed with TBST. The blots were visualized with chemiluminescence. Human β-actin was employed as an endogenous control.

### Gene expression data

The microarray datasets employed in this study was publicly available from ArraryExpress (http://www.ebi.ac.uk/arrayexpress/) of EBI and GEO (http://www.ncbi.nlm.nih.gov/gds/) of NCBI, including 6 independent cohorts of breast cancer (accession numbers: GSE15852 [14], GSE42568 [15], GSE4922 [16], GSE5847 [17], GSE23988 [18], E-TABM-158 [19]), 2 of lung cancer (E-MEXP-231 [20], GSE19804 [21]), 2 of prostate cancer (GSE6956 [22], GSE6919 [23]) and 2 of hepatocellular carcinoma (GSE14323 [24], GSE6764 [25]). The CEL files containing the raw data from each experiment were directly downloaded from the websites with particular accession number. Since RNA-seq is another popular method for genome-wide transcriptome profiling [26], one normalized RNA-seq dataset (GSE60788) of breast cancer was downloaded from GEO. Details of these datasets were summarized in Table 2 and Table 3.

**Table 2:** Five independent datasets from ArrayExpress and GEO website. Gene expression microarray datasets were normalized using RMA with package “affy”. Pearson correlation test was applied for examining the relationship of mRNA expression between C1orf63 and CDK10

Accession number	Array	Sample size	*r*	*P*
GSE4922	HG-U133A	249	0.292	2.86 × 10^−6^
GSE5847	HG-U133A	95	0.304	3.00 × 10^−3^
GSE23988	HG-U133A	61	0.327	1.00 × 10^−2^
E-TABM-158	U133AAofAv2	130	0.324	1.68 × 10^−4^
GSE60788	Illumina HiSeq 2000	55	0.521	4.57 × 10^−5^

**Table 3:** Eight independent datasets from ArrayExpress and GEO website. Gene expression microarray datasets were normalized using RMA with package “affy”. Student *t*-test was performed for examining the differential expression of C1orf63 between cases and controls of several cancers

Cancer	Accession number	Array	Sample size		Log-2 mRNA signal intensity (mean ± SD*)		*P*
			Control	Case	Control	Case	
Breast cancer	GSE42568	HG-U133_Plus_2	17	104	6.36 ± 0.54	7.57 ± 0.78	<0.001
	GSE15852	HG-U133A	43	43	9.49 ± 0.18	9.74 ± 0.21	<0.001
Lung cancer	E-MEXP-231	HG-U133A	9	49	7.83 ± 0.40	8.62 ± 0.65	0.001
	GSE19804	HG-U133_Plus_2	60	60	8.13 ± 0.73	8.41 ± 0.78	0.046
Prostate cancer	GSE6956	HG-U133A_2	20	69	9.44 ± 1.11	10.46 ± 0.36	<0.001
	GSE6919	HG_U95Av2	81	65	4.62 ± 0.24	4.67 ± 0.23	0.040
Hepatocellular carcinoma	GSE14323	HG-U133A_2	19	38	6.95 ± 0.46	7.82 ± 0.53	<0.001
	GSE6764	HG-U133_Plus_2	10	62	7.57 ± 0.81	8.45 ± 0.54	<0.001

*SD: standard deviation

In this paper, KM Plotter (http://kmplot.com/analysis/), a tool for the meta-analysis based biomarker assessment [27], including gene expression and survival data of more than 4000 breast cancer patients, was used to perform Kaplan Meier survival analysis to further assess the relationship between C1orf63 mRNA expression and RFS (relapse free survival)/OS (overall survival). Breast cancer patients were split by the median expression of C1orf63 into two groups, namely patients with high or low expression of C1orf63.

### Statistical analysis

Statistical analyses were performed using software SPSS (version 13.0) and R (version 3.0.2). The difference of C1orf63 protein expression between tumors and adjacent non-cancerous tissues were detected by Wilcoxon test, and the difference of online datasets retrieved C1orf63 mRNA expression between cases and controls of several cancer types included in this study were detected by Student *t*-test. Correlations between C1orf63 expression and clinicopathologic features were analyzed using chi-square test. Survival curves were calculated using the Kaplan–Meier method with log rank test. The Cox regression analysis was used to study the effects of C1orf63 expression on OS. OS (in months) was defined as the time from diagnosis to the date of last contact or of death from any cause. For gene expression microarray analyses, data were normalized using Robust Multi-array Analysis (RMA) with R-package “affy”. The normalized expression values (on a log-2 scale) of probes representing the same gene were averaged. Pearson’s correlation and Spearman’s rank correlation were applied for examining the relationship between C1orf63 and CDK10. *P* < 0.05 (two-tailed) was considered as statistically different.

## Results

### C1orf63 expression in breast cancer tissues and cell lines

**Fig. 1:**
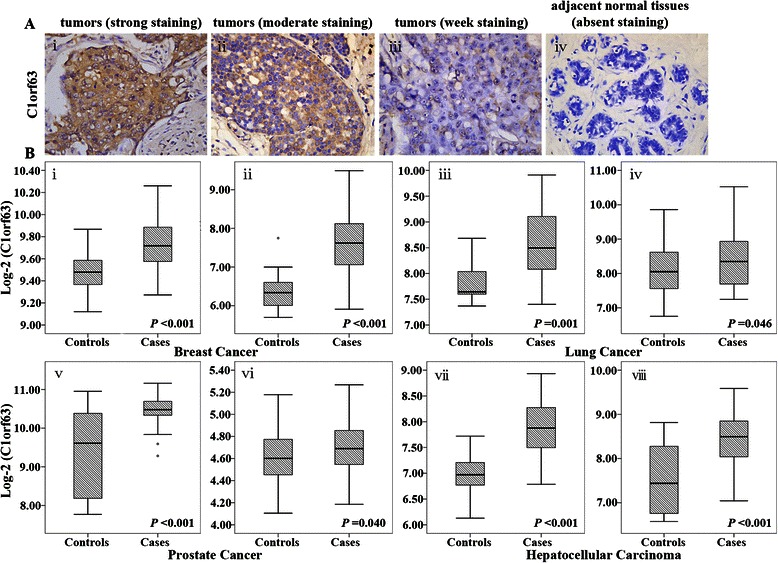
C1orf63 expression in cases and controls of several cancers .**a** IHC detected strong staining of C1orf63 in breast tumors (i, original magnification 400×), moderate staining of C1orf63 in breast tumors (ii, 400×), weak staining of C1orf63 in breast tumors (iii, 400×) and absent staining of C1orf63 in adjacent normal tissues (iv, 400×); **b** C1orf63 mRNA expression was significantly higher in cases than that in controls of several cancer, namely breast cancer (i, GSE15852; ii, GSE42568), lung cancer (iii, E-MEXP-231; iv, GSE19804), prostate cancer (v, GSE6956; vi, GSE6919) and hepatocellular carcinoma (vii, GSE14323; viii, GSE6764). *P* values were derived from student *t*-test

The tumor specimens and their matched adjacent non-cancerous tissues were collected from a group of 12 breast cancer patients to examine C1orf63 expression by IHC. As shown in Fig. 1A (i, ii, iii), C1orf63 protein was expressed primarily in the cytoplasm. We found 7 of the 12 primary tumors (58.3 %) expressed C1orf63 (Table 4), whereas 5 of the 12 tumors (41.7 %) had indistinctive expression of C1orf63. In contrast, all the adjacent normal tissues lacked elevated C1orf63 expression (Wilcoxon test: *P* < 0.001, Fig. 1A iv). Additionally, though analyzing the publicly available datasets, upregulation of C1orf63 mRNA expression was found in cases of breast cancer as well as other cancers, including lung cancer, prostate cancer and hepatocellular carcinoma (Table 3 and Fig. 1B), when compared to the relevant normal controls.

**Table 4:** C1orf63 scores detected by IHC in breast tumors and the adjacent normal tissues. 12 pairs of breast tumors and corresponding adjacent normal tissues were collected. The difference of C1orf63 expression between tumors and adjacent normal tissues were detected by Wilcoxon test

	C1orf63 scores (*n* = 8)		
	0	1 ~ 4	5 ~ 8
Tumors	0	5	7
Adjacent non-cancerous tissues	4	8	0

**Fig. 2:**
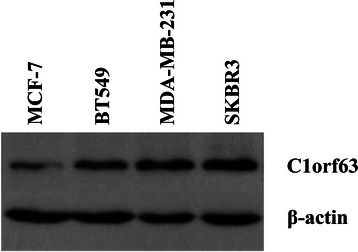
C1orf63 expression detected by Western blot in four human breast cancer cell lines including MCF-7, MDA-MB-231, SK-BR3, and BT549

We also performed western blotting to detect whether C1orf63 was expressed in breast cancer cells. Four human breast cancer cell lines, including the ER^+^/PR^+^ cell line MCF-7, ER^−^/PR^−^/Her-2^−^ cell lines BT549 and MDA-MB-231, and ER^−^/PR^−^/Her-2^+^ cell line SK-BR-3, were examined. As shown in Fig. 2, these cells have comparable levels of C1orf63 expression, regardless of receptor status.

### Relationship of C1orf63 with clinicopathologic features in a cohort of 182 breast cancer patients

To evaluate the relationship of C1orf63 expression with clinicopathological features, tumor sections from 182 primary breast cancer patients were immunostained to detect the expression of C1orf63, and these patients were subsequently divided into two groups according to their IHC scores: 44 (24.2 %) tumors expressing C1orf63 [C1orf63 (+) group] and 138 (75.8 %) tumors lacking C1orf63 expression [C1orf63 (−) group]. As shown in Table 1, no significant correlations were found between the expression of C1orf63 and the clinicopathological features collected in this study, including age, depth of invasion, lymph node metastasis and TNM stage. C1orf63 IHC score were also not associated to the expression of known breast cancer biomarkers including ER, PR or HER-2.

### Impact of C1orf63 expression on OS of breast cancer patients

**Fig. 3:**
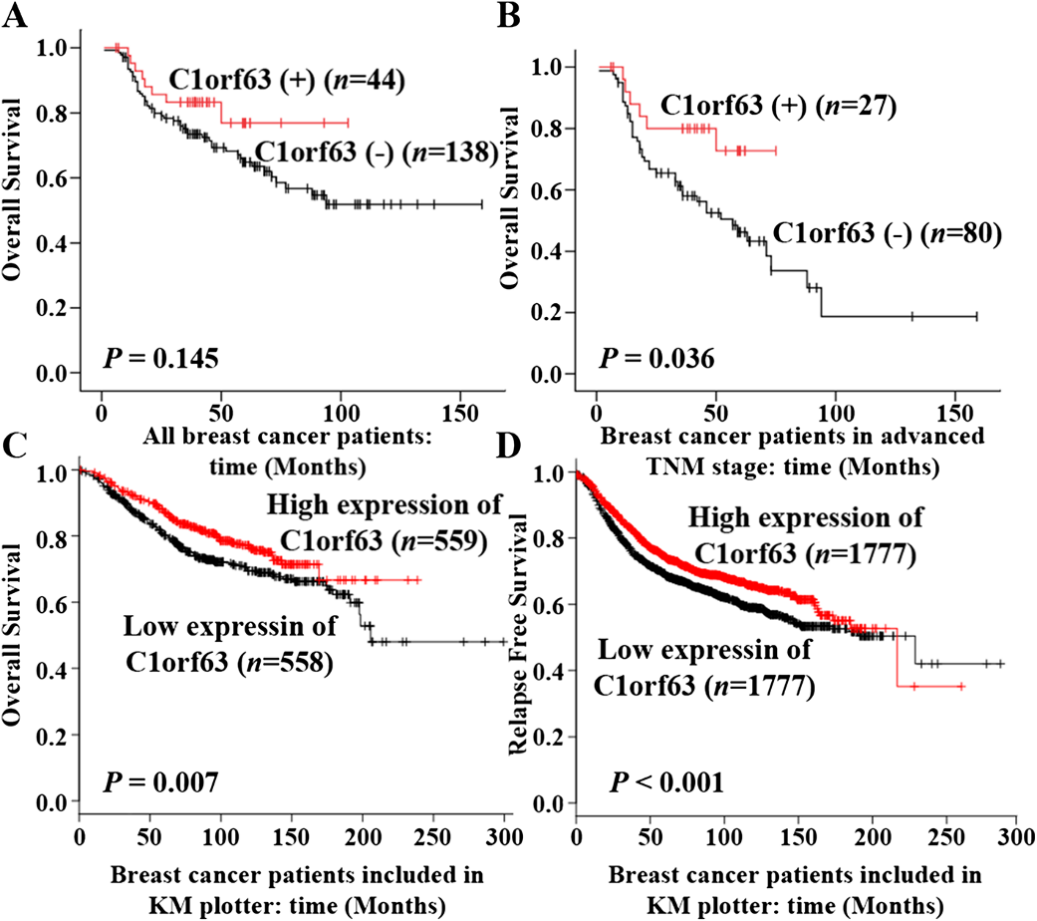
Kaplan-Meier survival analyses for assessment of the effect of C1orf63 expression on survival (log-rank test). **a** Effect of C1orf63 expression tested by IHC on OS in all the breast cancer patients; **b** Effect of C1orf63 expression tested by IHC on OS in breast cancer patients with advanced TNM stage (TNM III ~ IV stage); **c** Effect of C1orf63 mRNA expression on OS of breast cancer patients included in KM plotter; **d** Effect of C1orf63 mRNA expression on RFS of breast cancer patients included in KM plotter

**Fig. 4:**
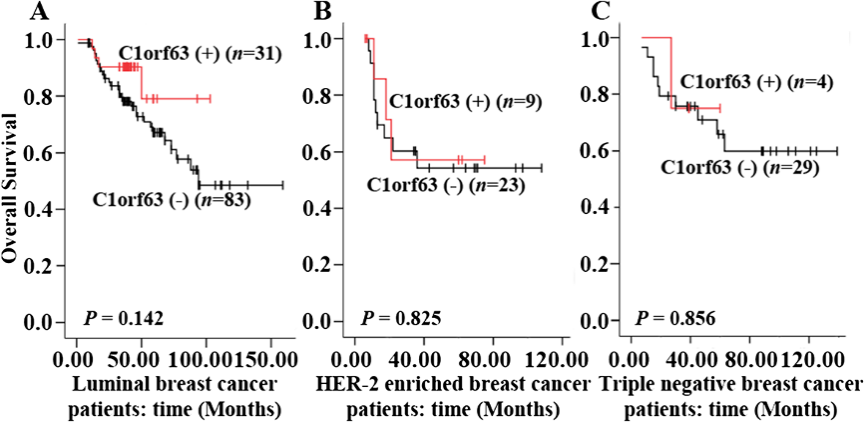
Kaplan-Meier survival analyses for assessment of the effect of C1orf63 expression in three different subtypes of breast cancer (log-rank test). Survival analyses of C1orf63 expression separately in luminal breast cancer(**a**), HER-2 enriched breast cancer (**b**), and triple negative breast cancer (**c**)

To examine whether the expression status of C1orf63 has any prognostic value for breast cancer, univariate and multivariate analyses using the Kaplan-Meier method and Cox regression were carried out. As shown in Table 5, of the 182 patients breast cancer, the OS rate in the C1orf63 (+) group was higher than that in the C1orf63 (−) group (3-year OS rates: 83.3 % vs 76.9 %; 5-year OS rates: 73.5 % vs. 64.9 %), but no significant difference was seen between these two groups (Log Rank *P* = 0.145, Fig. 3a), consistent with the result of univariate Cox regression (Table 6 left). KM Plotter tool was used to further assess the relationship between the mRNA expression of C1orf63 and RFS/OS of breast cancer patients. As shown in Fig. 3c and Fig. 3d, high expression of C1orf63 predicted a longer RFS and OS in breast cancer patients (*P* = 0.007; *P* < 0.001). The discordance between these two analyses suggests that the sample size of current IHC study (182 patients) may not be powerful enough to predict the outcome of the whole cohort. Since breast cancer is a heterogeneous disease with defined subtypes, we correlated IHC score of C1orf63 to the OS in individual subgroups, namely luminal (Fig. 4A), HER-2 enriched (Fig. 4B), and triple negative breast cancer patients (Fig. 4C), and no significant correlation was found. For the 182 breast cancer patients, log rank test also demonstrated that, depth of invasion (*P* < 0.001), lymph node metastasis (*P* < 0.001), advanced TNM stage (*P* < 0.001) and negative PR (*P* = 0.036), positive HER-2 (*P* = 0.028) were poor prognostic factors for OS. Patients with elder age (*P* = 0.074) or negative ER status (*P* = 0.242) had a shorter overall survival, but didn’t reach statistical significance (Table 5), which was consistent with the result of univariate Cox regression (Table 6 left).

**Table 5:** Overall survival related to clinicopathological features and biomarkers. 182 patients with breast cancer were included and the differences between these OS Rates were tested using the Kaplan–Meier method with log rank test

Variables	Patients	Events	OS rate (%)		*P*
			3-year (95 % CI)	5-year (95 % CI)	(log-rank)
Age, year					
≤ 60	153	45	78.3 (71.6, 85.0)	68.5 (60.1, 76.9)	0.074
> 60	29	13	62.0 (43.2, 80.8)	62.0 (43.2, 80.8)	
Depth of invasion					
T0 ~ T2	108	22	83.1 (75.7, 90.5)	78.0 (69.2, 86.8)	<0.001
T3 ~ T4	72	35	66.3 (55.3, 77.3)	54.3 (41.8, 66.8)	
Lymph node metastasis					
N0 ~ N1	93	17	87.8 (81.1, 94.5)	84.2 (76.2, 92.2)	<0.001
N2 ~ N3	87	40	63.3 (52.7, 73.9)	49.3 (37.0, 61.6)	
TNM stage					
I ~ II	74	9	92.9 (87.0, 98.8)	88.2 (79.8, 96.6)	
III ~ IV	107	49	63.6 (54.2, 73.0)	52.7 (41.9, 63.5)	<0.001
ER					
Negative	69	25	68.1 (56.7, 79.5)	63.6 (51.4, 75.8)	0.242
Positive	110	31	80.9 (73.5, 88.3)	69.3 (59.1, 79.5)	
PR					
Negative	107	39	65.8 (56.6, 75.0)	62.7 (52.9, 72.5)	0.036
Positive	72	17	91.3 (84.6, 98.0)	73.7 (60.8, 86.6)	
HER2					
Negative	108	27	80.9 (73.5, 88.3)	75.3 (65.9, 84.7)	0.028
Positive	70	29	71.1 (60.1, 82.1)	56.2 (43.5, 68.9)	
C1orf63					
Negative	138	50	73.5 (65.9, 81.1)	64.9 (56.1, 73.7)	0.145
Positive	44	8	83.3 (71.9. 94.7)	76.9 (61.0, 92.8)3

**Table 6:** Cox proportional hazard regression model analysis of OS in patients with breast cancer. 182 patients with breast cancer were included and the Cox regression analysis was used to study the effects of C1orf63 expression on overall survival (OS). HR, hazard ratio; 95 % CI: 95 % Confidence Interval

Variables	Univariate analysis		Multivariate analysis	
	HR (95 % CI)^*^	*P*	HR (95 % CI)^*^	*P*
Age, year	1.74 (0.94, 3.23)	0.079		
Depth of invasion	2.71 (1.58, 4.63)	<0.001		
Lymph node metastasis	3.81 (2.13, 6.84)	<0.001		
TNM stage	6.03 (2.91, 12.49)	<0.001	5.75 (2.76, 12.00)	<0.001
ER	0.73 (0.43, 1.24)	0.245		
PR	0.55 (0.31, 0.97)	0.040	0.54 (0.31, 0.96)	0.035
HER2	1.78 (1.05, 3.01)	0.031		
C1orf63	0.58 (0.27, 1.22)	0.152		

^*^HR, hazard ratio; 95 % CI: 95 % Confidence Interval

Next, multivariate analysis was performed to evaluate the implication of parameters including depth of invasion (T3 ~ T4), lymph node metastasis (N2 ~ N3), TNM stage (III ~ IV), PR negativity and HER-2 negativity on breast cancer prognosis (Table 6 right). We found that only TNM stage (HR: 5.75; 95 % CI: 2.76 ~ 12.00; *P* < 0.001) and PR (HR: 0.54; 95 % CI: 0.31 ~ 0.96; *P* =0.035) were independent prognostic indicators for breast cancer patients in our study.

### Impact of C1orf63 expression on OS of breast cancer patients with TNM III ~ IV Stages

Given the result of multivariate analysis mentioned above (Table 6 right), patients were further divided according to either TNM stage or PR levels, in order to analyze the impact of Clorf63 expression on OS in patients with different TNM stages or PR status. As shown in Table 7, tumors from 27 of 107 patients (25.2 %) with TNM III ~ IV stages expressed C1orf63, whereas tumors from the remaining 80 patients (74.8 %) lacked C1orf63 expression. Kaplan-Meier analysis revealed that patients in TNM III ~ IV stages with C1orf63 (+) tended to have a better prognosis than those without C1orf63 expression (3-year OS: 80.0 % vs. 58.0 %; 5-year OS: 72.7 % vs. 46.2 %; *P* = 0.036, Fig. 3B). In contrast, C1orf63 could not predict OS in patients with TNM early stage (TNM I ~ II stages, *P* = 0.432), or patients with PR negativity (*P* = 0.906) or PR positivity (*P* = 0.106) expression.

**Table 7:** Impact of C1orf63 expression on OS in different groups of breast cancer patients. 182 patients were divided according to either TNM stage or PR expression. There were 74 patients with TNM I ~ II stages, 107 patients with TNM III ~ IV stages, 74 patients with PR positive expression and 107 patients with PR negative expression. The differences of OS rates were tested using the Kaplan–Meier method with log rank test

Group	C1orf63 expression	Patients	Events	OS rate (%)^b^		*P* (log-rank)
				3-year (95 % CI)^c^	5-year (95 % CI)^c^	
TNM stages (I ~ II)	Negative	58	7	94.5 (88.4, 100.0)	83.4 (71.6, 95.2)	0.432
	Positive	16	2	87.5 (71.2, 100.0)	87.5 (71.2, 100.0)	
TNM stages (III ~ IV)	Negative	80	43	58.0 (46.8, 69.2)	46.2 (33.9, 58.5)	0.036
	Positive	27	6	80.0 (64.3, 95.7)	72.7 (53.1, 92.3)	
PR positive	Negative	50	16	87.2 (77.6, 96.8)	70.0 (55.5, 84.5)	0.106
	Positive	22	1	100^a^	83.3 (53.5, 100.0)	
PR negative	Negative	85	32	65.8 (55.4, 76.2)	62.1 (51.1, 73.1)	0.906
	Positive	22	7	65.0 (44.0, 86.0)	65.0 (44.0, 86.0)	

^a^ No patients died before the first three years, and the OS was 100 % while the 95 % CI were failed to calculate; ^b^OS rate: Overall survival rate; ^c^95 % CI: 95 % Confidence Interval

The relationship of C1orf63 expression with clinicopathological factors in patients with TNM III ~ IV stages was further evaluated using Cox regression. As shown in Table 8 left, the univariate analysis revealed that the C1orf63 (+) group tended to have a better prognosis than the C1orf63 (−) group (HR = 0.41; 95 % CI: 0.18 ~ 0.98; *P* = 0.044). Positive PR was also shown as a good prognosis factor for patients in TNM III ~ IV stages (HR = 0.52; 95 % CI: 0.27 ~ 0.97; *P* = 0.039). However, no significant difference was observed regarding other clinicopathological features. To examine whether C1orf63 was an independent prognosis factor for patients with TNM III ~ IV stages, multivariate analysis was performed. It demonstrated that both C1orf63 expression (Table 8 right, HR: 0.41; 95 % CI: 0.17 ~ 0.97; *P* = 0.042) and PR (HR: 0.51; 95 % CI: 0.27 ~ 0.95; *P* = 0.035) were independent prognostic factors for patients in this subgroup.

**Table 8:** Cox regression analysis of breast cancer patients with TNM III ~ IV stages. 107 breast cancer patients with TNM III ~ IV stages were included and the Cox regression analysis was used to study the effects of C1orf63 expression on overall survival (OS). HR, hazard ratio; 95 % CI: 95 % Confidence Interval

Variables	Univariate analysis		Multivariate analysis	
	HR (95 % CI)*	*P*	HR (95 % CI)*	*P*
Age, year	1.50 (0.76, 2.95)	0.239		
Depth of invasion	1.37 (0.74, 2.52)	0.32		
Lymph node metastasis	1.26 (0.59, 2.72)	0.549		
ER	0.69 (0.39, 1.24)	0.218		
PR	0.52 (0.27, 0.97)	0.039	0.51 (0.27, 0.95)	0.035
HER2	1.62 (0.91, 2.90)	0.102		
C1orf63	0.41 (0.18, 0.98)	0.044	0.41 (0.17, 0.97)	0.042

*HR, hazard ratio; 95 % CI: 95 % Confidence Interval

### Association between C1orf63 and CDK10 in breast cancers

**Fig. 5:**
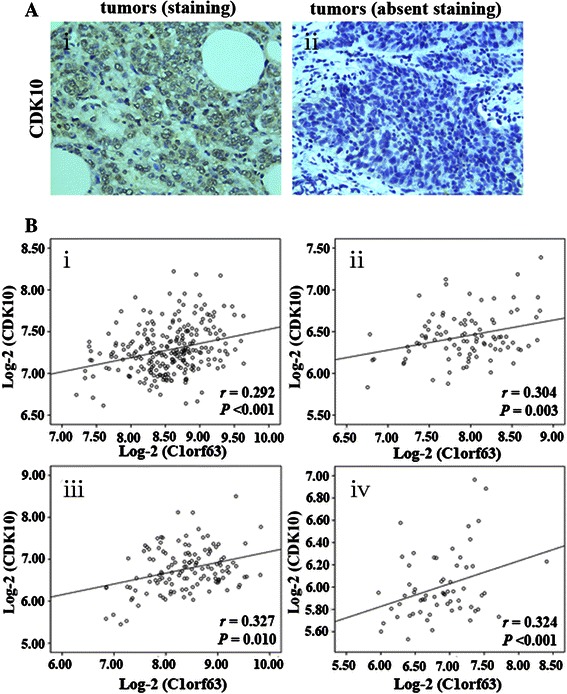
C1orf63 expression in human breast tissues and its correlation with CDK10. IHC detected staining of CDK10 in tumors (a, *i*) and absent staining for CDK10 in tumors (a, *ii*); CDK10 was significantly correlated with C1orf63 in patients with breast cancer from datasets E-GEOD-4922 (b, *i*), E-GEOD-5847 (b, *ii*), E-GEOD-23988 (b, *iii*) and E-TABM-158 (b, *iv*). The Y-axis and X-axis respectively represented the mRNA expression of CDK10 and C1orf63 on the log-2 scale

CDK10 has been shown to play a role in cellular progression as well as a known prognostic factor predicting better outcome for breast cancers. Given the suggested role of C1orf63 on cell cycle exit [8], and its capability to predict better prognosis for breast cancers (Fig. 3B and Table 8), we thus examined the relationship between C1orf63 and CDK10. We firstly took advantage of four publicly available microarray datasets, each including a cohort of patients with breast cancer, to evaluate whether mRNA expression of C1orf63 could be related to that of CDK10. As shown in Fig. 5B, for mRNA expression, C1orf63 was positively correlated with CDK10, and the RNA-seq dataset also displayed a significant correlation between these two genes (*r* = 0.521, *P* < 0.001; Table 2). Further, CDK10 protein expression was examined by IHC. As shown in Fig. 5Ai, CDK10 primarily expressed in the nucleus and the relationship between the IHC scores of CDK 10 and C1orf63 was consistent with that of their mRNA expressions, which demonstrated that C1orf63 expression was positively correlated with CDK10 (*r*_s_ = 0.391; *P* < 0.001).

## Discussion

Aberrations of chromosome 1 are one of the most frequently detected alterations in a variety of cancers [28–32]. There are numerous putative candidate oncogenes located on chromosome 1, e.g., *NEGR1* (1p31.1), *JTB* (1q21), *CKS1B* (1q21.2), *CHD1L* (1q12), *SHC1* (1q21) and *KIF14* (1q32.1) [33–35]. Besides, 1p36 deletion has been reported to be associated with carcinogenesis, and contain genes such as *CHD5* (1p36.31), *CAMTA1* (1p36.31-p36.23), *KIF1B* (1p36.22), and *CASZ1* (1p36.22). This specific location suggests C1orf63 might be related to initiation and development of cancer. However, the function of C1orf63 has been rarely described. It is demonstrated that C1orf63 protein is interacted with CLK3 (CDC-like kinase 3) and CLK2 (CDC-like kinase 2) protein [36], both of which are involved in protein phosphorylation and regulation of RNA splicing. It was stated that AKT activation controls cell survival to ionizing radiation by phosphorylating CLK2 [37]. As a CLK2-interacting protein, C1orf63 might participate in these processes. Additionally, an association between a nonsynonymous SNP (rs1043879) in C1orf63 and ESR (erythrocyte sedimentation rate), which is a marker of several serious disease such as infection, autoimmune disorder, and malignancy [38], was revealed by a genome-wide association study with unclear biological significance. However, all the speculations about C1orf63 still need further study. Current study is the first to focus on the implication of C1orf63 in breast cancers.

The genes with abnormal expression hold important clinical implications as prognostic markers and/or targets for cancer therapy. According to our results, C1orf63 seems to have dual functions. The tumor-promoting function of C1orf63 in the initialization of breast cancer was suggested not only by the higher IHC score of C1orf63 in breast tumors when compared to adjacent non-cancerous tissues, also by the higher mRNA expression of C1orf63 in breast tumor vs. normal controls through analyzing several breast cancer gene expression datasets. More importantly, the tumor- promoting function of C1orf63 might not be limited to breast cancer, because gene expression dataset analysis showed that C1orf63 expression was also elevated in several other cancer types, including lung, prostate and hepatocellular carcinoma. Current study also indicated that the tumor-promoting function of C1orf63 might not involve ER, PR or HER-2, as no significant correlation was observed between the expression of C1orf63 and these biomarkers in either breast cancer tissues or cell lines. KM Plotter analysis of breast cancer patients showed that elevated mRNA expression of C1orf63 is significantly correlated with both longer RFS (*P* < 0.001) and betetr OS (*P* = 0.007), suggested a tumor suppression function of C1orf63. Most probably limited by the sample size of current IHC study, the C1orf63 IHC score failed to correlate with OS of all the breast cancer patients, but is capable of predicting a better prognosis for breast cancer patients in TNM III ~ IV stages, strongly indicating that C1orf63 could also act as a tumor suppressor, especially in the advanced stage of breast cancer. Until now, the relationship of C1orf63 with cancer remains largely unknown. Pils et al. [39] demonstrated that C1orf63 mRNA was differently expressed between epithelial ovarian cancer patients and controls, but the evidence is still limited. We provided the first evidence for the implication of C1orf63 in breast cancer tumorigenesis and progression, and demonstrated that the function of C1orf63 was complicated.

C1orf63 has been suggested to function in typical tumor initiation event as cell cycle exit and maintenance of quiescent state of cells [8]. Many chemotherapeutical drugs such as Paclitaxel and 5-FU elicit their anti-tumor activities through forcing cancer cells staying quiescently [40, 41]. Whether C1orf63 could enhance the efficacy of therapeutical drugs via keeping cells in a quiescent state and thus predict a better outcome of cancer patients is unknown but a potential mechanism. Similar to C1orf63, the dual functions have been observed for many proteins, such as SRSF1 (serine/arginine-rich splicing factor 1). SRSF1 is a proto-oncogene that is overexpressed in many different cancers. However, increased SRSF1 expression in primary human fibroblasts could ultimately triggers oncogene-induced senescence via stabilizing p53 [42]. Even so, the mechanism under the seemingly reversible action of C1orf63 still needs further study.

Recent studies have shown that CDK10 is a potential tumor suppressor in breast cancers, and CDK10/Ets2/c-RAF signaling has been demonstrated as an important determinant of breast cancer resistance to endocrine therapy [43]. Since C1orf63 might be involved in cell cycle exit, we thus correlated the IHC score of CDK10 to that of C1orf63 in the same cohort, and detected that higher C1orf63 expression was positively associated with enhanced CDK10 expression, suggesting that C1orf63 probably function in a mechanism involving CDK10. Further research is needed to detect the underlying mechanism.

## Conclusions

C1orf63 expression was supposed to be an early event of breast cancer oncogensis. It served as a favourable and independent prognostic marker for patients with breast cancer in TNM III ~ IV stages, suggesting C1orf63 might elicit two different functions involved in the oncogenesis and progression of breast cancer. Moreover, its positive correlation with CDK10 suggests that C1orf63 might be involved in cell cycle progression. Further work are warranted to better understand the potential function of C1orf63 in cancer pathogenesis.

## Abbreviations

C1orf63: Chromosome 1 open reading frame 63
CDK10: Cyclin-dependent kinase 10
ER: Estrogen receptor
PR: Progesterone receptor
HER-2: Human epidermal growth factor receptor 2
IHC: Immunohistochemistry
OS: Overall survival
RFS: Relapse free survival
